# A single-cell platform for reconstituting and characterizing fatty acid elongase component enzymes

**DOI:** 10.1371/journal.pone.0213620

**Published:** 2019-03-11

**Authors:** Alexis A. Campbell, Kenna E. Stenback, Kayla Flyckt, Trang Hoang, M Ann DN Perera, Basil J. Nikolau

**Affiliations:** 1 Roy J Carver Department of Biochemistry, Biophysics and Molecular Biology, Iowa State University, Ames, Iowa, United States of America; 2 Center for Metabolic Biology, Iowa State University, Ames, Iowa, United States of America; 3 NSF-Engineering Research Center for Biorenewable Chemicals, Iowa State University, Ames, Iowa, United States of America; 4 W.M. Keck Metabolomics Research Laboratory, Iowa State University, Ames, Iowa, United States of America; Universite Paris-Sud, FRANCE

## Abstract

Fatty acids of more than 18-carbons, generally known as very long chain fatty acids (VLCFAs) are essential for eukaryotic cell viability, and uniquely in terrestrial plants they are the precursors of the cuticular lipids that form the organism’s outer barrier to the environment. VLCFAs are synthesized by fatty acid elongase (FAE), which is an integral membrane enzyme system with multiple components. The genetic complexity of the FAE system, and its membrane association has hampered the biochemical characterization of FAE. In this study we computationally identified *Zea mays* genetic sequences that encode the enzymatic components of FAE and developed a heterologous expression system to evaluate their functionality. The ability of the maize components to genetically complement *Saccharomyces cerevisiae* lethal mutants confirmed the functionality of *ZmKCS4*, *ZmELO1*, *ZmKCR1*, *ZmKCR2*, *ZmHCD* and *ZmECR*, and the VLCFA profiles of the resulting strains were used to infer the ability of each enzyme component to determine the product profile of FAE. These characterizations indicate that the product profile of the FAE system is an attribute shared among the KCS, ELO, and KCR components of FAE.

## Introduction

Two primary characteristics affect the chemo-physical properties of fatty acids that enable them to fulfill diverse biological functions, the length of the alkyl-chain, and the number and geometric isomerism of carbon-carbon double bonds in the alkyl-chain. In eukaryotic organisms, such as plants, fatty acids are initially assembled as fully saturated acyl-chains, and subsequently carbon-carbon double bonds are created in the alkyl chain by an aerobic process catalyzed by specific desaturases, which remove hydrogen atoms from adjoining carbon atoms [1]. Therefore, the processes of initial fatty acid assembly from simple precursors is important in determining the carbon chain-length of these molecules. Plants express three fatty acid assembly processes, two of which are catalyzed by acyl carrier protein (ACP)-dependent, Type II fatty acid synthase (FAS) systems, which are localized in plastids [2] and mitochondria [3,4]. The third system is an ER-localized fatty acid elongase (FAE) [5,6], which uses preexisting fatty acyl-CoAs as substrates for further elongation. The former two systems assemble the bulk of fatty acids produced within a cell, and these are primarily between 14- and 18-carbon chain-lengths, and the FAE system produces very long chain fatty acids (VLCFAs) of ≥20 carbon atoms.

All three fatty acid assembly systems utilize the same biochemical mechanism, iterative reaction cycles of condensation-reduction-dehydration-reduction. Apart from the distinct organelle localizations of the three assembly systems, three additional features distinguish the FAE system from FAS: 1) in the FAE system the pantetheine group that carries the intermediates of the growing fatty acyl chain is Coenzyme A (CoA); 2) the FAE system is an integral membrane-bound system localized to the endoplasmic reticulum; and 3) FAE initiates the elongation process by using pre-existing (C16 or C18) fatty acyl-CoAs.

As exemplified by the lethal consequence of mutations that affect VLCFA biosynthesis, these acyl-chains participate in vital cellular functions, specifically associated with cellular sphingolipid pools [7–10]. These vital functions include protein trafficking, membrane structure, lipid secondary messaging, and protection from environmental stresses [11,12]. For example, VLCFAs are incorporated into the skin of mammals to provide a protective permeability barrier. As exemplified by the death of *elovl1* and *elovl4* null mutant mice that cannot produce VLCFAs, these components contribute a critical functionality to the skin’s normal function and structure [9,13,14]. Similarly, aerial plant surfaces are covered with a permeability barrier (i.e., the cuticle) composed of a heterogeneous mixture of VLCFAs and their derivatives, which acts as a barrier against non-stomatal water loss, and biotic as well as abiotic stresses [15]. The metabolic fate of VLCFAs in plants is not limited to surface lipids, they are also components of the pollen coat, suberin, sphingolipids, glycerolipids, phospholipids and triacylglycerols [12,16].

Plants have generated and maintain a large degree of biochemical and genetic redundancy within the FAE system. Two distinct non-homologous enzyme families catalyze the initial condensation reaction: 1) the Arabidopsis FAE1-like, 3-ketoacyl-CoA synthases (KCS-type enzymes); and 2) the ELONGATION DEFECTIVE-LIKEs (ELO-type enzymes). These two enzymes catalyze the same chemical reaction, creating a new C-C bond, and this reaction is thought to be the chain-length determining and possibly rate-limiting reaction of the FAE system [17,18].

The second reaction of the FAE cycle is catalyzed by 3-ketoacyl-CoA reductase (KCR); two highly homologous KCR paralogs, *gl8a* (*ZmKCR1*) and *gl8b* (*ZmKCR2*), have been characterized in maize, with partially redundant functions [19–21]. In contrast, Arabidopsis contains only one functional isoform of the KCR enzyme [22]. The remaining FAE components, the 3-hydroxyacyl-CoA dehydratase (HCD) and the enoyl-CoA reductase (ECR), are encoded by single-copy genes in Arabidopsis and yeast [23–26] and these are hypothesized to have broad substrate specificity, capable of generating the complete gamut of VLCFA chain-lengths [18, 27–29].

In this study, we have utilized heterologous expression in *Saccharomyces cerevisiae* to identify four additional catalytic components of the *Zea mays* FAE system using the strategy of genetic complementation of yeast mutant strains as an assay to identify gene functionality. The maize FAE system is not only of interest because of its importance to global agriculture, but because of the unique complexity that maize presents by the functional redundancy of the ELO, KCS, and KCR components. Biochemical characterization of the fatty acid profiles of the complemented yeast strains demonstrate that the maize ELO, KCS, and KCR paralogs contribute different fatty acid profiles, indicating that these components contribute to the product specificity of the overall FAE systems.

## Materials and methods

### Identification of maize FAE components

With the exception of the KCR component [19–21], all other maize FAE components were putatively identified by sequence homology with experimentally validated Arabidopsis and yeast FAE enzymatic components. *ZmKCS* (GenBank accession AFW81175.1; GRMZM2G393897) and *ZmELO* (GenBank accession CM007647.1; GRMZM2G037152) were identified based on sequence homology to a characterized Arabidopsis KCS (KCS9; At2g16280) [30] or yeast, human, and putative Arabidopsis ELOs respectively. Both ORFs were PCR amplified from the genomic DNA of *Zea mays* inbred line B73. *ZmKCR1* and *ZmKCR2* were previously genetically identified and molecularly isolated as the *Glossy8a* and *Glossy8b* loci, respectively [19–21]. The maize HCD homolog (*ZmHCD*; GenBank accession DR817981.1, GRMZM2G151087) was identified from the maize EST assembly at PlantGDB (www.plantgdb.org) based on its sequence homology to the yeast (*PHS1*; *YJL097w*) [23] and Arabidopsis HCD (*PASTICCINO2*; AT5G10480) [25]. The maize ECR component (*ZmECR*; GenBank accession AQK90310.1, GRMZM2G481843) was identified by its homology to the yeast (*TSC13*; *YDL015c*) [27] and Arabidopsis ECR (*CER10*; AT3G55360) [26]. *ZmECR* was independently shown to be encoded by the genetically defined *Glossy26* locus, which is required for the normal deposition of cuticular waxes [31]. Both *ZmHCD* and *ZmECR* cDNAs were isolated by RT-PCR from the *Zea mays* inbred line B73.

### Phylogenetic analysis of the maize FAE enzymes

Neighbor-joining phylogenetic trees were constructed for each of the FAE components, and these included homologous sequences identified from *Arabidopsis thaliana*, *Zea mays*, *Solanum lycopersicum*, *Sorghum bicolor*, *Oryza sativa*, *Saccharomyces cerevisiae*, and *Homo sapiens*. Homologs were identified by BLASTP [32] analysis with the maize, Arabidopsis, and yeast protein sequences using Ensembl [33]. Sequences were aligned with MUSCLE [34], using the MEGA7 software package, and the bootstrap consensus tree was inferred from 1000 replicates. Phylogenetic trees were rooted with the appropriate Arabidopsis fatty acid synthase protein components i.e., 3-ketoacyl-ACP synthase I, II, III (KASI, encoded by AT5G46290; KASII, encoded by AT1G74960; KASIII, encoded by AT1G62640), 3-ketoacyl-ACP reductase (KAR, encoded by AT1G24360), 3-hydroxyacyl-ACP dehydratase (HD, encoded by AT2G22230 and AT5G10160), and enoyl-ACP reductase (ER, encoded by AT2G05990).

### Construction of yeast expression cassettes

All yeast expression cassettes used the galactose-inducible *GAL1* promoter to control the heterologous expression of maize FAE components [35]. Using the Gateway® cloning system ORFs encoding the *ZmKCS*, *ZmELO*, *ZmHCD* and *ZmECR* components were cloned into the high-copy episomal plasmids, pAG426 (*URA3*), pAG423 (*HIS3*) or pAG424 (*TRP1*) (Invitrogen, Carlsbad, CA) [35] (S1 Table). “Entry” clones were constructed by cloning PCR amplification products generated with the appropriate primer pairs (S2 Table). *ZmKCR1* and *ZmKCR2* were each cloned into pYX043 (an integrative yeast shuttle vector carrying the *LEU2* marker), using the *Eco*RI-*Sal*I and *Kpn*I-*Xba*I restriction sites, respectively. All recombinant yeast shuttle vectors were confirmed by DNA sequencing, and were maintained in *E*. *coli* TOP10 or DH5α cells (Invitrogen, Carlsbad, CA), propagated in Luria Bertani (LB) media with the appropriate antibiotics.

### Site-directed mutagenesis

During PCR-based construction of the *ZmECR*/pAG424 cassette a random mutation within the *ZmECR* coding sequence was generated, as an A-to-T transversion that caused a serine to cysteine change at position 283 of the ECR protein sequence (GenBank Protein Accession ACF86977). This mutated construct, *ZmECR*(p.Ser283Cys)/pAG424, was used as a negative control in the yeast genetic complementation experiments. The transversion was corrected by QuikChange mutagenesis (Stratagene, La Jolla, CA) using primer ECRcorrA2T (S2 Table) and confirmed by sequencing, to generate *ZmECR*/pAG424.

### Yeast strains and media

Yeast cultures were grown according to standard procedures [36,37]. The parental strains, BY4741, BY4743, CEN.RO16, WDAM006, and α-D273 (genotype information available in S3 Table), were maintained in rich YPD (Yeast Peptone Dextrose) media and grown in YP-galactose (YPGal) media as inductive conditions. Yeast strains carrying mutations in the endogenous FAE component genes were obtained from Open Biosystems (Huntsville, AL) and are listed in S3 Table. These strains and all derivatives that carry the gene-disrupting KanMX4 cassette were selected by growth on media containing 200 μg/mL Geneticin (G418; Invitrogen, Carlsbad, CA). Yeast strains carrying maize expression cassettes were selected by their ability to grow on minimal medium (SD) without the appropriate amino acid or nucleobase (e.g., uracil). Expression of maize FAE components was induced with the inclusion of 2% galactose in YPGal medium. Counter-selection of *URA3* carrying vectors was done in the presence of 100 μg/mL 5-fluoroorotic acid (5-FOA; US Biological, Swampscott, MA) in SD media.

### Yeast genetics

Plasmids were transformed into yeast using a standard lithium acetate transformation protocol [38]. Homologous recombination-induced integration of pYX043-based cassettes were conducted by transforming yeast strains with *Bst*XI-digested linearized plasmids. For the purpose of generating and maintaining the synthetically lethal *scelo2*, *scelo3* double mutant strain, a *ScELO3*-expression vector was constructed by cloning the *ScELO3* sequence with its native promoter sequence in the pAG416 (low copy, *URA3*) plasmid backbone using In-Fusion cloning (Takara Bio USA, Inc., Mountain View, CA). Restriction sites and primers were designed to remove the promoter region of the vector backbone. The resulting plasmid (carrying P_*ELO3*_-*ELO3*) was transformed into a diploid strain that was heterozygous mutant at both *scelo2* and *scelo3* loci. The resulting strain was sporulated, enabling the recovery of the *scelo2*, *scelo3* double mutant, which episomally expressed P_*ELO3*_-*ELO3*. This was genetically confirmed by the inability of the strain to grow on 5-FOA counter-selection media, and molecularly confirmed with PCR.

Sporulation of diploid strains was performed according to Enyenihi and Saunders [39] with the following modifications. Sporulation was induced by growing the diploid strains in supplemented liquid medium (1% potassium acetate, 0.005% zinc acetate, and 1x appropriate amino acid drop-out supplements) at 25°C for 7–14 days. Tetrad-containing suspensions were mixed with an equal volume of 1 mg/ml Zymolyase-100T (United States Biologicals, Salem, MA) in 1M sorbitol solution, and the mixture was incubated at 37°C for 15 minutes. Tetrads were dissected using a Nikon Eclipse 50i Dissection microscope (Nikon Instruments Inc., Elgin, IL), and spores were “pulled” from the partially digested ascus by micromanipulation and placed onto the appropriate selective induction media (SD) plates. In experiments testing the functional expression of maize FAE components, spores were placed on SD-media containing galactose.

### Strain growth analysis

Growth of all yeast strains were assessed in synthetic complete (SC) or SD media, supplemented with the appropriate auxotrophic need, and expression of maize genes was induced with galactose. Strains were grown in shake flasks or using a BioTek microplate reader (BioTek Instruments Inc., Winooski, VT), maintained at 30°C with constant shaking, and growth was monitored by measuring OD600 every 30 minutes. Doubling times were calculated using Gen5 data analysis software (https://www.biotek.com/products/software-robotics-software/gen5-microplate-reader-and-imager-software/), and statistical evaluations were carried out using JMP Pro 9.0 (SAS Institute Inc. Cary, NC), using Student’s *t* tests to identify statistical rigor. Raw data is available at DOI 10.25380/iastate.7635962.

### Fatty acid analysis

Fatty acids were extracted from yeast cell pellets following saponification with barium hydroxide [21,40], with the following modifications. Yeast cells were collected by centrifugation and the cell pellets were flash frozen in liquid nitrogen and lyophilized. Although each yeast strain grew with different growth profiles, we took take care to ensure that for each series of complementation experiments were grown in parallel and all genotypes were taken at the same time-point; i.e., at 42-hours post-induction, when all strains were at stationary phase of growth.

Between 5 and 10 mg of dry cell pellet and a known amount of nonadecanoic acid, as an internal standard, was homogenized with acid-washed glass beads (425–600 μm, Sigma-Aldrich, St. Louis, MO), and treated with a solution of 10% (w/v) barium hydroxide at 110°C for 24-hours. Upon acidification, the saponified fatty acids were recovered by extracting with hexane. Following methylation and silylation, fatty acid methyl esters were analyzed by GC-MS or GC-FID [40,41]. Statistical rigor of the data were evaluated by 3–11 replicates per experiment, specific values are listed in figure legends and raw fatty acid accumulation data is available at DOI 10.25380/iastate.7635959. Statistical analyses were carried out using JMP Pro 13.0 (SAS Institute Inc., Cary, NC). The metabolite abundance data were evaluated using Tukey Honest Significant Difference (HSD) to evaluate statistical rigor.

## Results and discussion

### Phylogenetic analyses of the FAE components

**Fig 1:**
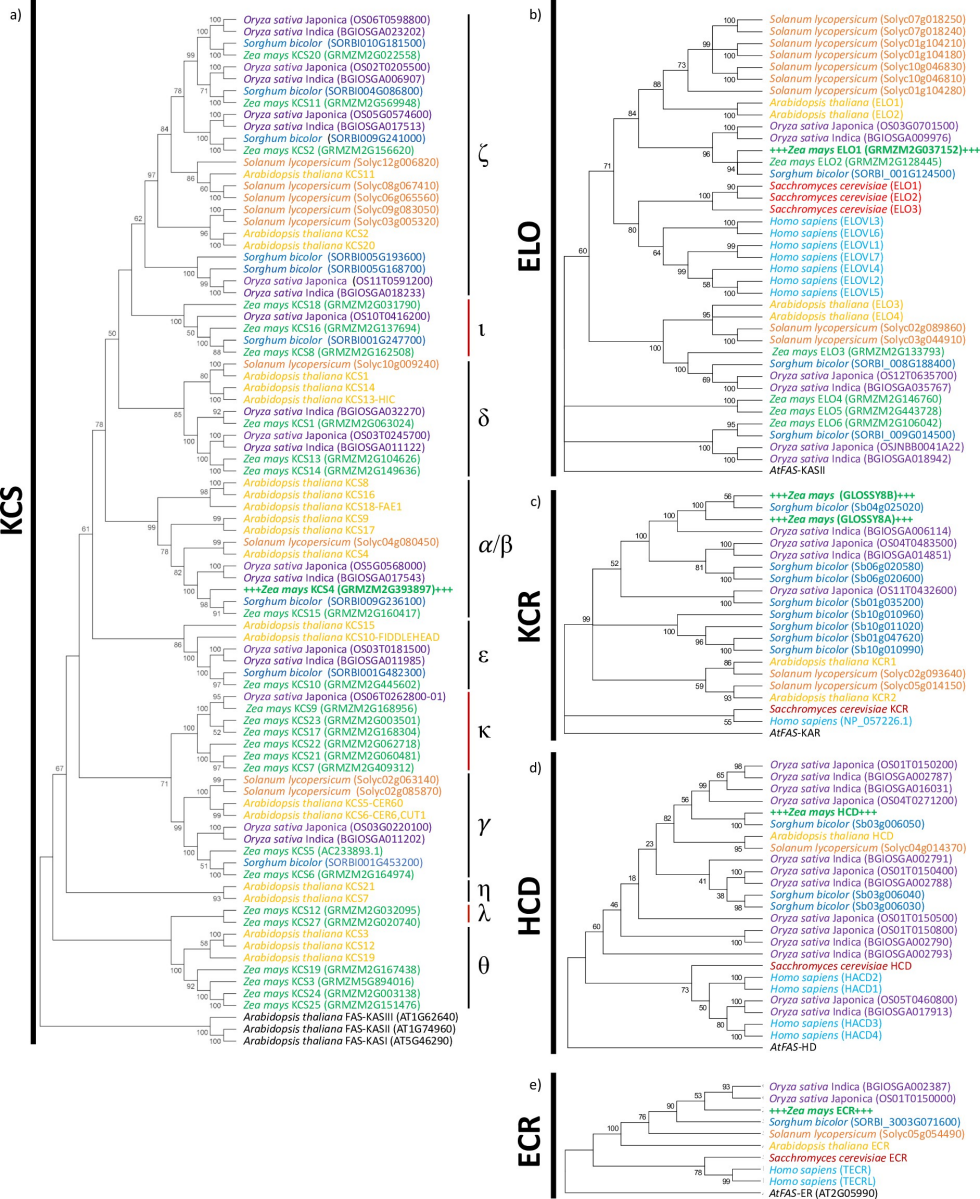
Phylogenetic relationships among FAE enzyme components based on the comparison of the amino acid sequences. The MEGA7 package was used for the construction of the phylogenetic neighbor-joining tree and bootstrapped 1000 times. The trees were rooted to the appropriate Arabidopsis fatty acid synthase component. **a)** KCS: putative KCS candidates were separated into clades (α-λ). **b)** ELO **c)** KCR **d)** HCD **e)** ECR.

Fig 1 shows the phylogenetic relationships among the five enzymatic components of the FAE system. These analyses compare representative sequences from mammals, yeast, and dicotyledonous and monocotyledonous plants. Fig 1A shows the relationships among the 26 maize candidate KCS enzymes, which were classified based on the eight Arabidopsis KCS subclasses, previously labeled as clades α to θ [42]. These analyses indicate that, every clade defined by the Arabidopsis homologs, except clade η, contains a maize homolog. In addition, based upon bootstrap values that are greater than 60, three three new clades are identified (ι, κ and λ), which appear to be monocot-specific; clade ι contains three maize, one rice, and one sorghum homologs, clade κ contains six maize and one rice homologs, and clade λ contains two maize homologs. In the molecular complementation experiments conducted in this study we chose the maize KCS encoded by the gene GRMZM2G393897 (*ZmKCS4*), which is classified as belonging to the phylogenetically mixed α/βclade.

Similar sequence-based analyses identified six maize *ELO* homologs. These candidates were further classified based on the occurrence of four conserved amino acid motifs (i.e., HX_2_HH, KX_2_EX_2_DT, HX_2_MYX_2_YY, TX_2_QX_2_Q) that have been experimentally characterized as being functionally important with yeast ELOs [43] (Fig 1B). Furthermore, because prior studies have established that functional ELO enzymes contain 5–7 transmembrane domains [44], the maize sequences were also computationally analyzed for the occurrence of transmembrane domains [45]. Based upon these analyses (Table 1) we have identified the protein encoded by the GRMZM2G037152 locus as the most likely candidate to encode an ELO-like condensing enzyme, which we term, *ZmELO1*. The other five candidates were not considered for further experimental validation because they only partially retain the yeast and human ELO-defined conserved motifs (Table 1).

**Table 1:** Amino acid motif and membrane domain analyses of ELO enzymes.

ELO Protein Characteristics					
	Number of Predicted Transmembrane Domains	Amino Acid Motifs			
	TMHMM	KXXEXXDT	HXXHH	HXXMYXYY	TXXQXXQ
**ScELO1**	5	yes	yes	yes	yes
**ScELO2**	7	yes	yes	yes	yes
**ScELO3**	6	yes	yes	yes	yes
**HsELOVL1**	6	yes	yes	yes	yes
**HsELOVL2**	7	yes	yes	yes	yes
**HsELOVL3**	7	yes	yes	yes	yes
**HsELOVL4**	7	yes	yes	yes	yes
**HsELOVL5**	7	yes	yes	yes	yes
**HsELOVL6**	6	yes	yes	yes	yes
**HsELOVL7**	5	yes	yes	yes	yes
**ZmELO1 (GRMZM2G037152)**	7	yes	yes	yes	yes
**ZmELO2 (GRMZM2G128445)**	3	XXXEXXDT	no	no	XXXQXXQ
**ZmELO3 (GRMZM2G133793)**	6	XXXEXXDT	no	no	XXXQXXQ
**ZmELO4 (GRMZM2G146760)**	10	no	XXXHH (2)	no	no
**ZmELO5 (GRMZM2G443728)**	10	no	XXXHH (2)	no	no
**ZmELO6 (GRMZM2G106042)**	0	no	XXXHH	no	no

Fig 1C shows the phylogenetic relationships among the previously characterized maize (encoded by *Glossy8a* and *Glossy8b* [19–21]), yeast [46] and Arabidopsis [22] KCRs, and compares their sequences to other KCR homologs computationally identified by their shared sequence similarities. These analyses reveal the common occurrence of the putative catalytic motif (SX_16_YX_3_K) [19,22] and the NADH-binding motif (GX_3_GXGX_3_AX_3_AX_2_G), which is commonly conserved in the broader short chain dehydrogenases/reductase superfamily of enzymes [47]. In addition, 11 of the 19 analyzed KCR proteins contain the N-terminal dilysine ER-retention signal, which is expected for type I ER-localized proteins [48].

These phylogenetic analyses establish that KCR enzymes divide into three separate clades, specific to monocotyledonous plants, dicotyledonous plants, and yeast and mammals. This finding therefore, indicates that the evolutionary history of this FAE component is ancient and precedes the divergence of Plantae from Fungi and Animalia. In addition, like maize, many plant species contain multiple KCR homologs, which appear to have arisen post-speciation, and while some of these duplications have retained the KCR catalytic function, as is the case for maize [19–21], in other species, such as Arabidopsis, only one duplicated homolog has retained this catalytic capability [22].

Similar sequence-based phylogenetic analyses using yeast, human and Arabidopsis sequences identified putative maize, rice, sorghum, and tomato HCD and ECR homologs (Fig 1D and 1E). Apart from rice, which appears to encode 6 or 7 HCD homologs and sorghum, which encodes three HCD homologs, these two catalytic components are encoded by single-copy genes in other sequenced plant genomes. Moreover, these two enzymatic FAE components classify into distinct Plantae and Fungi/Animalia clades, and do not show clear division between monocotyledonous and dicotyledonous plants, indicating that they arose prior to the divergence of monocots and dicots.

### Genetic complementation of yeast mutants lacking FAE components

**Fig 2:**
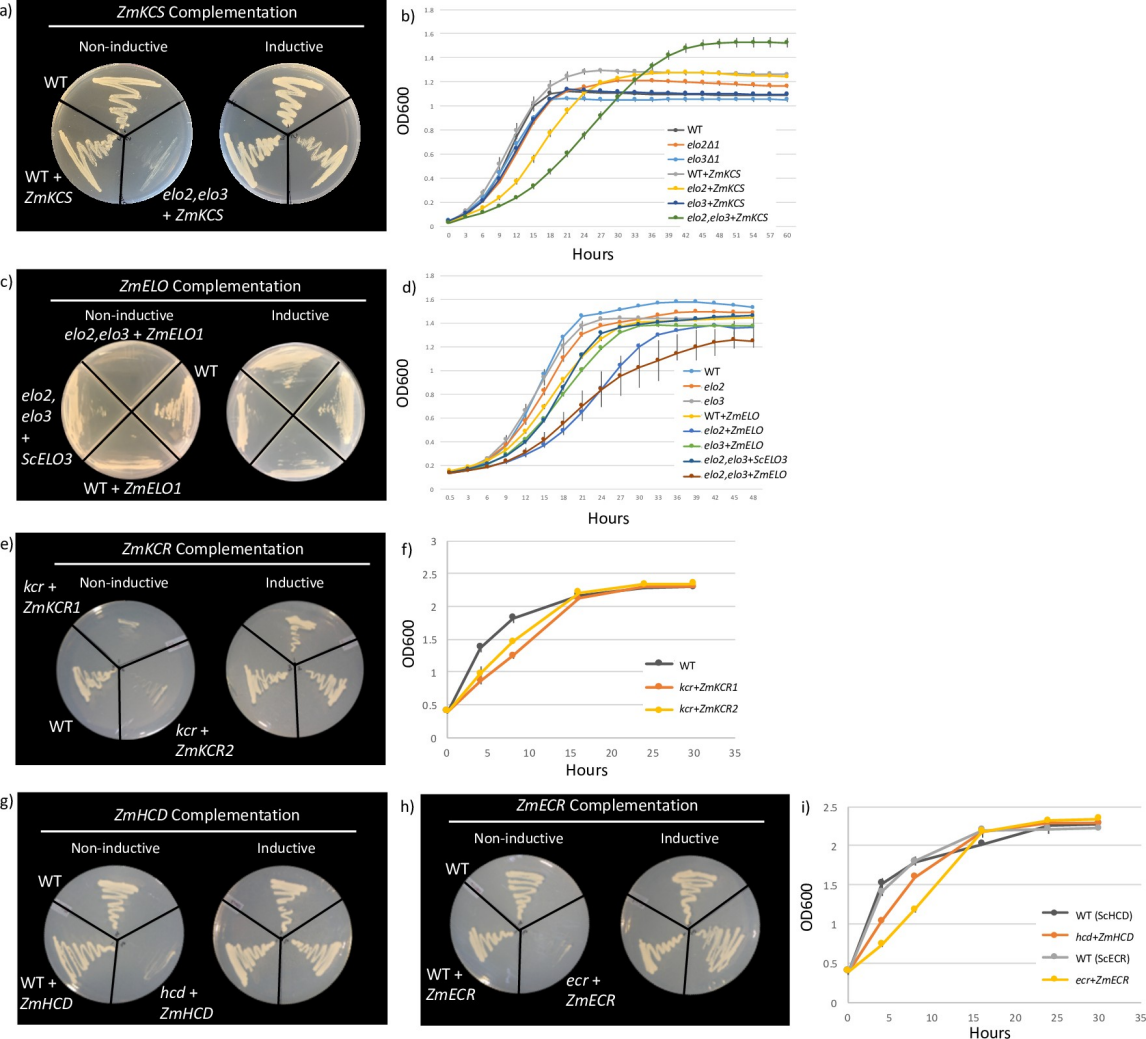
Genetic complementation of yeast FAE mutants by maize components under non-inductive and inductive conditions and growth analysis. **a)** Recovered spores from an individual tetrad of the heterozygous *scelo2*, *scelo3* with *ZmKCS4*. 1) wild-type (BY4742); 2) *scelo2*, *scelo3* with *ZmKCS4*; and 3) wild-type with *ZmKCS4*. **b)** Growth analysis for *ZmKCS4* overexpression strains and mutants. **c)** *ZmELO1* complementation following 5-FOA counter-selection. 1) wild-type (BY4741); 2) wild-type with *ZmELO1*; 3) *scelo2*, *scelo3* with *ZmELO1; and* 4) *scelo2*, *scelo3* with *ScELO3* (P_*ELO3*_-*ELO3*). **d)** Growth analysis for wild-type, *ZmELO1* overexpression strains, and yeast mutants. **e)** Recovered spores from an individual tetrad of the heterozygous *sckcr* strain containing *ZmKCR1* or *ZmKCR2*. Spores shown are from 1) *sckcr* with *ZmKCR1*; 2) *sckcr* with *ZmKCR2*; and 3) CEN.RO16 (wild-type). **f)** Growth analysis for wild-type and *ZmKCR1* and *ZmKCR2* complementing strains. **g)** Recovered spores from an individual tetrad of the heterozygous *schcd* strain containing *ZmHCD*. 1) wild-type (BY4742); 2) *schcd* with *ZmHCD*; and 3) wild-type with *ZmHCD*. **h)** Recovered spores from and individual tetrad of the heterozygous *scecr* with *ZmECR*. 1) wild-type (W303); 2) *scecr* with *ZmECR*; and 3) wild-type with *ZmECR*. **i)** Growth analysis for wild-type and *ZmHCD* and *ZmECR* complementing strains.

The catalytic functionality of maize FAE components (*ZmKCS4*, *ZmELO1*, *ZmKCR1*, *ZmKCR2*, *ZmHCD* and *ZmECR*) were individually evaluated based on their ability to complement yeast strains lacking the respective endogenous function. The yeast mutant strains used in this study carry disruption knock-out alleles, which are either lethal (i.e., *scelo2*, *scelo3* double mutant [49]*; schcd* [23]; and *scecr* [27]) or near lethal (*sckcr* [46]). Each maize ortholog was individually expressed in these yeast strains under the transcriptional control of the *GAL1* promoter, and in every case growth of the resulting yeast strains was observed only when they were grown on galactose-containing media, inducing gene expression from the *GAL1* promoter (Fig 2A, 2C, 2E, 2G and 2H).

As an additional control, all complemented strains were in parallel compared to strains that expressed maize FAE components in a wild-type yeast strain, which carried functional alleles of the endogenous yeast FAE components. In the case of the functional complementation with the *ZmECR* component, an additional control experiment was with the use of the ZmECR (p.Ser283Cys) mutant. This point mutation is at a residue that appears to be crucial, because the resulting strain is inviable, and suggests that residue p.Ser283 is critical to catalysis.

**Fig 3:**
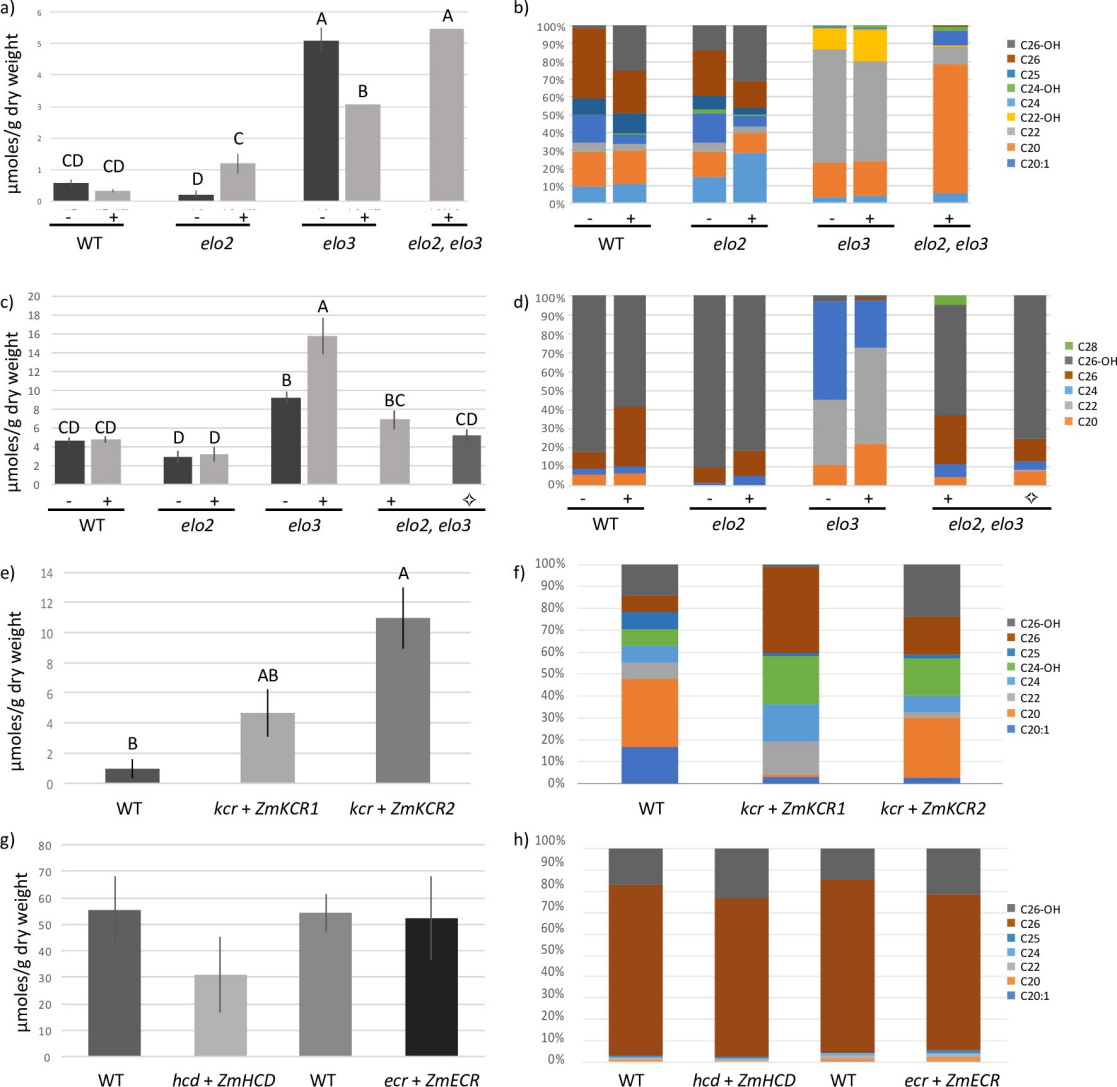
Quantitative totals of VLCFAs and molar percentage of total VLCFA product pools. **a,b)** *ZmKCS4* (n = 6), **c,d)** *ZmELO1* (n = 6) where (◇) indicates the presence of the maintenance plasmid (P_*ELO3*_-*ELO3*), **e,f)** *ZmKCR1* and *ZmKCR2* (n = 3), and **g,h)** *ZmHCD* (n = 8 for control n = 10 for complementing strain) and *ZmECR* (n = 8 for control n = 11 for complementing strain). Differing letters indicate statistically significantly different yields based on Tukey HSD (p value<0.05). All strains were analyzed using GC-MS except *ZmELO1*, which was analyzed by GC-FID. Yeast strain is indicated under graphs, were (-/+) indicated the absence and presence of the maize gene respectively for **a-d**.

Because of the genetic and biochemical redundancy in the enzymes that catalyze the condensation reaction between an acyl-CoA and malonyl-CoA, which forms a new carbon-carbon bond in each catalytic FAE cycle, the analysis with the ZmKCS4 and ZmELO1 homologs were more complex than the complementation experiments with the other FAE components. Specifically, in yeast three gene homologs (*ScELO1*, *ScELO2* and *ScELO3*) encode the enzyme that catalyzes the condensation reaction in the yeast FAE cycle, but only the latter two are crucial for VLCFA biosynthesis [50,51]. Namely, yeast strains that carry individual *scelo1*, *scelo2*, or *scelo3* knockout mutant alleles are viable, but the *scelo2*, *scelo3* double mutant is lethal [49]. Therefore, to dissect the ability of the *ZmKCS4* and *ZmELO1* to complement this deficiency, we evaluated the quantitative and qualitative changes in the VLCFA profiles of the complemented *scelo2*, *scelo3* double mutant strains, and compared these to the wild-type and each *scelo2* and *scelo3* single mutant, in the presence and absence of the maize complementing gene (Fig 3A–3D).

The synthetic lethality associated with the *scelo2*, *scelo3* double mutant can be complemented by the overexpression of either *ZmKCS4* or *ZmELO1* (Fig 2A and 2C), although the growth of the recovered strains is significantly compromised relative to the wild-type strain (Fig 2B and 2D and Table 2). The growth of the strains genetically complemented with either the ZmELO1 or ZmKCS4 condensing enzymes is slower than the wild-type strains. In contrast however, the strains complemented by the maize KCR, HCD, or ECR components display growth curves that are similar to the wild-type (Fig 2B, 2D, 2F and 2I). All maize FAE components (except *ZmHCD*) were also expressed individually in the wild-type background, resulting in the overexpression of each catalytic FAE functionality. In the case of the *ZmKCS4* and *ZmELO*1 components, we also evaluated the effect of overexpression on the growth of the viable, individual *scelo2* and *scelo3* mutant strains (Fig 2B and 2D). With the exception of the overexpression of *ZmELO1* in the *scelo2* mutant, which doubled the culture’s doubling time, these latter manipulations did not significantly affect the growth rate of the cultures (Table 2).

**Table 2:** Doubling time (hours) and standard error (SE) for *ZmELO1* and *ZmKCS4* expressing strains. Different letter superscripts indicate statistically significantly different doubling times based on Tukey HSD, n = 6 (p value<0.05).

Doubling Time														
Yeast Strain	WT		WT + gene		*elo2*		*elo2 +* gene		*elo3*		*elo3* + gene		*elo2*, *elo3 +* gene	
Maize Gene	Hours	SE	Hours	SE	Hours	SE	Hours	SE	Hours	SE	Hours	SE	Hours	SE
*ELO1*	3.82^C^	0.02	5.3^A,B,C^	0.10	4.81^A,B,C^	0.08	7.15^A^	0.12	4.09^B,C^	0.16	5.56^A,B,C^	0.13	6.80^A,B^	0.61
*KCS4*	3.64^F^	0.07	3.72^E,F^	0.16	4.59^C^	0.02	5.56^B^	0.13	4.05^D,E^	0.054	4.28^C,D^	0.02	7.47^A^	0.11

### VLCFA profiles in yeast strains genetically complemented with maize FAE components

The fatty acid profiles of each yeast mutant strain that was rescued by the expression of individual maize FAE components were compared to the wild-type strains, profiling the detectable fatty acids, from 12 to 28 carbon chain length. As expected, changes in the profiles of the products of *de novo* FAS (i.e., ≤18-carbon chain length), which accounts for >90% of the fatty acids in these strains, were only subtely affected by the genetic manipulations of FAE components (S1 Fig); the exception being the overexpression of the *ZmKCS4* in the *scelo3* background, and the overexpression of *ZmKCR1* or *ZmKCR2* in the *sckcr* background,. In contrast, the genetic manipulations of the KCS, ELO, and KCR components caused significant changes in the products of FAE (i.e., ≥20-carbon chain length), and these are illustrated in Fig 3.

The expression of either ZmKCS4 or ZmELO1 induced two different effects on VLCFA profiles. The *ZmKCS4* complementation of the *scelo2*, *scelo3* double mutant strain induced a nearly 10-fold increase in the levels of VLCFAs as compared to the wild-type strain, and 20:0 accounts for the majority of the additional VLCFAs that accumulate (Fig 3A and 3B). This contrasts with the effect of overexpressing *ZmKCS4* in the wild-type background or from that observed when it’s expressed in the two individual *scelo2* and *scelo3* single mutants. Each of these latter *ZmKCS4-*overexpressing strains generate quantitatively and qualitatively distinct VLCFA profiles. For example, overexpressing *ZmKCS4* in the wild-type strain does not affect the total VLCFA content, but does change the VLCFA profile, inducing the accumulation of larger quantities of 2-hydroxy-FAs (i.e., 2-hydroxy-26:0). However, overexpression of *ZmKCS4* in viable *scelo2* or *scelo3* single mutant strains, induced inverse effects on VLCFA accumulation between the two strains. Specifically, *ZmKCS4* overexpression in the *scelo2* background induced increased accumulation of VLCFAs as compared to the parental mutant strain; whereas in the *scelo3* background, the yield of VLCFA is reduced. These differences are primarily due to the individual *scelo2* or *scelo3* mutations, as the composition of the VLCFA profiles remain unaltered by the overexpression of *ZmKCS4*.

The VLCFA profile and product titer of the *ZmELO1*-complemented, *scelo2*, *scelo3* double mutant is near identical to the wild-type and to the strain overexpressing *ZmELO1* in a wild-type background (Fig 3C and 3D). This similarity in the behavior of the *ZmELO1* expressing strain is also observed when *ZmELO1* is overexpressed in the *scelo2* lacking strain, but not in the *scelo3* lacking strain. Namely, the *scelo3* mutant with or without the overexpression of *ZmELO1* accumulates larger quantities of VLCFAs, particularly 20:0, 22:0 and 24:0.

The genetic complementation of the *sckcr* mutant strain by the expression of either *ZmKCR1* or *ZmKCR2* produced significantly different FAE product profiles, and these are each distinct from the wild-type strain (Fig 3E and 3F); the former profile is qualitatively different from the wild-type, whereas the latter profile is quantitatively different. Specifically, the VLCFA content in the *ZmKCR1*-rescued strain is not statistically different from the wild-type (p-value 0.277), but the VLCFA profile of this strain is distinct from the wild-type, producing larger amounts of non-hydroxylated 22:0, 24:0, and 26:0 fatty acids. The *ZmKCR2*-rescued strain hyperaccumulates VLCFAs by 10-fold over the wild-type strain (p-value 0.009), but the resulting VLCFA profile resembles the wild-type strain. In contrast to the strains that are genetically complemented by the expression of either *ZmKCR1* or *ZmKCR2*, the overexpression of either of these two maize components in a wild-type background produces only subtle quantitative or qualitative changes in the VLCFA profiles. The exception is the production of 28:0 by the *ZmKCR2* overexpressing strain, which is a VLCFA product that is undetectable in the absence of the maize gene (S2 Fig).

The expression of ZmHCD or ZmECR either in a wild-type background (i.e., overexpression of these two components) or in the appropriate mutant background (i.e., genetically complemented strains) generates VLCFA profiles that are indistinguishable from the wild-type strains (Fig 3G and 3H and S3 Fig). These results indicate that these two maize components have broad acyl-chain length substrate specificities, being able to respectively dehydrate 3-hydroxyacyl-CoA and reduce enoyl-CoA intermediates, irrespective of the chain-length that is generated by the of the yeast FAE system.

## Conclusion

Several attributes of FAE that generates VLCFA molecules contribute to the inability to biochemically characterize this system. These include the fact that a) FAE is an integral membrane system associated with the ER, making its biochemical isolation and characterization more difficult; b) there has been confusion as to the chemical nature of the acyl substrate needed by FAE [52]; c) in most tissues (with the exception of seeds of the Brassicaceae family) products of FAE account for a small proportion of the fatty acids in the tissue; and d) there are genetic and biochemical redundancies in the individual components of the FAE system [21,49,53].

In this study we have demonstrated a yeast-based platform that can be used to characterize the *in vivo* properties of the individual FAE enzymatic components, as they impact the product profile of the FAE system. Collectively, the series of genetic complementation experiments validate the use of yeast as a heterologous expression platform for the characterization of the maize FAE system. Exemplary of this validation, the platform was used to functionally characterize the role of maize *KCS4*, *ELO1*, *KCR1*, *KCR2*, *HCD* and *ECR* components on determining the VLCFA product profile of the FAE system. This platform ultimately has the capability of evaluating the genetic diversity among the KCS, ELO, and KCR components, and providing a direct means to functionally evaluate how the FAE system is assembled and regulated to generate a diversity of end products. The experiments described herein indicate that there are two major enzymatic determinants that govern the product profile of the FAE system: the first and second reaction of the four-reaction cycle that constitutes the iterative biochemistry of fatty acid elongation. In maize, the first reaction can be catalyzed by up to 33 different enzymes (26 KCS type and 6 ELO type isozymes) and the second reaction can be catalyzed by two KCR isozymes. This diversity of component enzymes suggests that maize has the potential of expressing a large number of different FAE complexes differing in the KCS, ELO and KCR components.

Exemplary of plants, the maize genome encodes two families of enzymes that may catalyze the formation of new carbon-carbon bonds during each FAE-catalyzed elongation cycle, the KCS-class of enzymes, and the ELO-type enzymes. The contribution of this potential biochemical redundancy to the product specificity of the plant FAE system is unknown. The archetypal KCS enzyme was identified by the analysis of the Arabidopsis *fae1*-mutant, which expresses a deficiency in the ability to sequentially elongate 18:1 to 20:1 and 22:1 during seed development [54–56]. Subsequent bioinformatic and genetic [57] studies identified 21 KCS-coding genes in Arabidopsis, some of which appear to be associated with the deposition of the cuticle [30,58,59]. Our computational search identified 26 homologs in the maize genome. The maize KCS homologs were classified relative to a phylogenetic system that was initially defined by the Arabidopsis KCS homologs [57] leading to the identification of three new monocot-specific clades of KCS enzymes (ι, κ and λ).

In contrast to KCS, much less is known about the plant ELO family of enzymes. The ELO-type enzymes were initially identified in *S*. *cerevisiae*, which expresses three homologs [23,49–51]. Genetic-based characterizations identified that *ELO2* and *ELO3* are responsible for elongating 16- and 18-carbon fatty acids to chain-lengths of up to 26 carbons [49]. Moreover, simultaneously knocking out both of these genes is synthetically lethal, due to the inability to assemble the appropriate ceramide with a VLCFA [49]. Computationally we identified and characterized the sequences of six maize ELO-like enzymes. We utilized the yeast genetic platform to begin the molecular characterization of one of the maize ELOs and evaluated its role in contributing to the product specificity of the FAE system, as compared to KCS. The experiments presented herein establish, for the first time, that plant ELO-homologs contribute catalytic functionality to FAE, and hence experimentally establish that plant genomes encode biochemically redundant proteins capable of catalyzing the condensation reaction of the FAE cycle.

The expression of either *ZmELO1* or *ZmKCS4* can rescue the viability of the yeast strain that lacks both *scelo2* and *scelo3* functions. The fatty acid profiles of these rescued strains indicate that, whereas the expression of *ZmELO1* reconstituted the VLCFA profile to that of the wild-type strain, the expression of *ZmKCS4* generated a VLCFA profile that is distinct from the wild-type. This difference in the biochemical outcomes between the *ZmELO1* and *ZmKCS4* is also apparent when these maize genes are expressed in the wild-type yeast background, and in the *scelo2* or *scelo3* single mutant backgrounds. Both the individual *scelo2* and *scelo3* mutants are viable in the absence of the maize genes, but they generate different VLCFA profiles from the wild-type, and the expression of *ZmKCS4* in these strains generates uniquely distinct VLCFA profiles.

Collectively, these finding indicate that despite the large evolutionary structural differences between the maize and the yeast FAE proteins, the yeast system has sufficient flexibility to accommodate the plant components. However, because the *ZmKCS4*-expressing strains generate VLCFA profiles that are distinct from the “normal” yeast profiles, whereas the *ZmELO1*-expressing strains generate near wild-type VLCFA profiles, we suggest that the yeast FAE system better accommodates the latter enzyme. This can be rationalized by the fact that ELO homologs share greater structural similarity with the yeast FAE components, as compared to KCS, facilitating the interactions between the other three enzymatic components of the yeast FAE system. Assuming each KCS or ELO homolog may have different substrate specificities, this complexity provides plants with the ability to generate diverse VLCFA profiles. We further infer therefore, that interactions between the ELO/KCS components and the other three components are significant in determining the product profile of FAE.

Indeed the expression of the two highly homologous maize KCR enzymes (encoded by the *GLOSSY8A* (*ZmKCR1*) and *GLOSSY8B* (*ZmKCR2*) genes [19–21]), further substantiates this supposition. The differential, but partially redundant functionalities of ZmKCR1 and ZmKCR2 is indicated by the fact that *glossy8a* mutations reduce the seedling leaf cuticle, whereas *glossy8b* mutations have no such effect on this trait; however, the *glossy8a*, *glossy8b* double mutant is embryo-lethal [21]. This genetic redundancy in maize contrasts with the situation in Arabidopsis, which expresses a single functional KCR and a non-functional homolog [22].

The genetic complementation of the near-lethal *sckcr* [46] mutant strain by the expression of either of the two maize KCR homologs establishes that both are capable of catalyzing the reduction of the 3-ketoacyl-CoA intermediates of the FAE cycle. However, as revealed by the VLCFA profiles of the two complemented yeast strains, the two ZmKCRs confer distinct biochemical outcomes. Specifically, the *ZmKCR1*-complemented strain expresses similar quantities of VLCFAs as the wild-type, but these products are of distinct chain-length distribution from the wild-type. The *ZmKCR2*-complemented strain in contrast, produces larger quantities of VLCFAs, but with much more subtle change in the VLCFA profile.

A possible explanation for this variance maybe associated with different substrate specificties of each ZmKCR homolg. However, a more complex model may need to be invoked when one considers that the two ZmKCR homologs vary from each other by only 11 conservative substitutions among 326 residues [21]. A potential model may be that ZmKCR2 better associates with the endogenous yeast ELO-containing FAE system, and thus generates a VLCFA titer and compositional profile that is more similar to the wild-type strain. In contrast, ZmKCR1 prefers to associate with KCS-containing FAEs, which are not present in yeast, and thus its expression generates a very distinct VLCFA titer and profile. This discriminatory interaction explanation is consistent with the *in planta* chemotypes that were characterized with the *glossy8a* (*zmkcr1*) and *glossy8b* (*zmkcr2*) mutants of maize [21]. Specifically, the *glossy8a* mutant that only expresses *ZmKCR2* primarily affects the VLCFA-derived cuticular lipids, and does not affect the VLCFAs that are associated with the ceramide lipids, whereas the *glossy8b* mutant does not affect the deposition of cuticular lipids, but primarily reduces the VLCFAs associated with ceramides [19,21].

In the context of the two types of FAE systems that can be envisioned in maize (one that utilizes KCS and one that utilizes ELO condensing enzymes), these KCR-associated attributes may indicate that ZmKCR1 and ZmKCR2 have differential product targeting effects by selectively preferring to associate with one or the other of the FAE systems. Based on the fact that the ELO-containing FAE system in yeast preferentially generates the VLCFAs that are used to assemble the ceramide moiety of sphingolipids [60], and if this attribute extrapolates to maize, one can infer that ZmKCR2 preferentially associates with ELO-containing FAE systems that generate ceramides, whereas ZmKCR1 prefers to associate with the KCS-containing FAE systems, generating the bulk of the VLCFAs that are destined for the cuticle.

The ability of *ZmHCD* and *ZmECR* to complement the respective mutations in the yeast FAE components indicates that these maize enzymes can also associate with the other yeast FAE components and enable the successful completion of the VLCFA biosynthetic process. In contrast to the alterations of the VLCFA profiles that result from replacing the yeast ELO and KCR components with maize homologs, the replacement of the yeast HCD and ECR components with the maize homologs did not change the resulting VLCFA profiles. We conclude therefore that the enzyme components that catalyze the 3^rd^ and 4^th^ reactions of the FAE cycle are not significant determinants of the product specificity of the FAE system.

Although the plant FAE system has previously been isolated as an apparent complex [61–63] due to its low abundance, and as an integral membrane enzyme system, its structural organization is still mysterious. It may therefore be possible to use this platform to determine how the FAE system is organized in the ER membrane. Moreover, in light of the fact that plants, exemplified by maize, possess a high level of potential biochemical and genetic redundancy in the assembly of a large number of distinct types of FAEs, the platform described here has the potential to explore the structure-function relationships among the different catalytic components, in heterologously reconstituted and assembled system.

## Supporting information

S1 Fig Total FAS generated fatty acids and product pools. Quantitative totals of FAS products and molar percentage of total FAS product pools for **a, b)** *ZmKCS4* (n = 6), **c, d)** *ZmELO1* (n = 6) where (◇) indicates the presence of the maintenance plasmid (P_*ELO3*_-*ELO3*), **e, f)** *ZmKCR1* and *ZmKCR2* (n = 3), and **g, h)** *ZmHCD* (n = 8 for control n = 10 for complementing strain) and *ZmECR* (n = 8 for control n = 11 for complementing strain). Differing letters indicate statistically significantly different yields based on Tukey HSD (p value<0.05). All strains were analyzed using GC-MS except *ZmELO1*, which was analyzed by GC-FID. Yeast strain is indicated under graphs, were (-/+) indicated the absence and presence of the maize gene respectively for **a-d**.
(PDF)

S2 Fig Total FAS and FAE generated fatty acids and product pools for WT and WT with *ZmKCR1* or *ZmKCR2*. **a, c, e, g)** WT with empty vector (pYX043, n = 3) and WT with *ZmKCR1* (n = 4). **a)** Quantitative totals of FAS products; **b)** Molar percentage of total FAS product pools; **c)** Quantitative totals of VLCFAs; and **d)** Molar percentages of totally VLCFAs product pools. **b, d, f, h)** WT with empty vector (pYES2) and WT with *ZmKCR2* (n = 5). **b)** Quantitative totals of FAS products; **d)** Molar percentage of total FAS product pools; **f)** Quantitative totals of VLCFAs; and **h)** Molar percentages of total VLCFA product pools.
(PDF)

S3 Fig Total FAS and FAE generated fatty acids and product pools for WT with the empty vector (pYX043) and WT with *ZmECR*. **a)** Quantitative totals of FAS products; **b)** Molar percentage of total FAS product pools; **c)** Quantitative totals of VLCFAs; and **d)** Molar percentages of totally VLCFAs product pools. Strains were analyzed with replicates of n = 4 for WT with the empty vector (pYX043) and n = 5 for WT with *ZmECR*.
(PDF)

S1 Table List of plasmids used within this study along with relevant characteristics. (PDF)

S2 Table Primers used to construct FAE reconstitution plasmids and yeast strains. (PDF)

S3 Table Yeast genotype and strain information for this study. (PDF)
