# Supplementary material for: A single-cell platform for reconstituting and characterizing fatty acid elongase component enzymes
Source: PLoS One. 2019 Mar 11;14(3):e0213620. doi: 10.1371/journal.pone.0213620 (PMC6411113; doi:10.1371/journal.pone.0213620)
Supplement: S1 Fig — Quantitative totals of FAS products and molar percentage of total FAS product pools for a, b) ZmKCS4 (n = 6), c, d) ZmELO1 (n = 6) where (◇) indicates the presence of the maintenance plasmid (PELO3-ELO3), e, f) ZmKCR1 and ZmKCR2 (n = 3), and g, h) ZmHCD (n = 8 for control n = 10 for complementing strain) and ZmECR (n = 8 for control n = 11 for complementing strain). Differing letters indicate statistically significantly different yields based on Tukey HSD (p value<0.05). All strains were analyzed using GC-MS except ZmELO1, which was analyzed by GC-FID. Yeast strain is indicated under graphs, were (-/+) indicated the absence and presence of the maize gene respectively for a-d. (PDF) [file pone.0213620.s001.pdf]

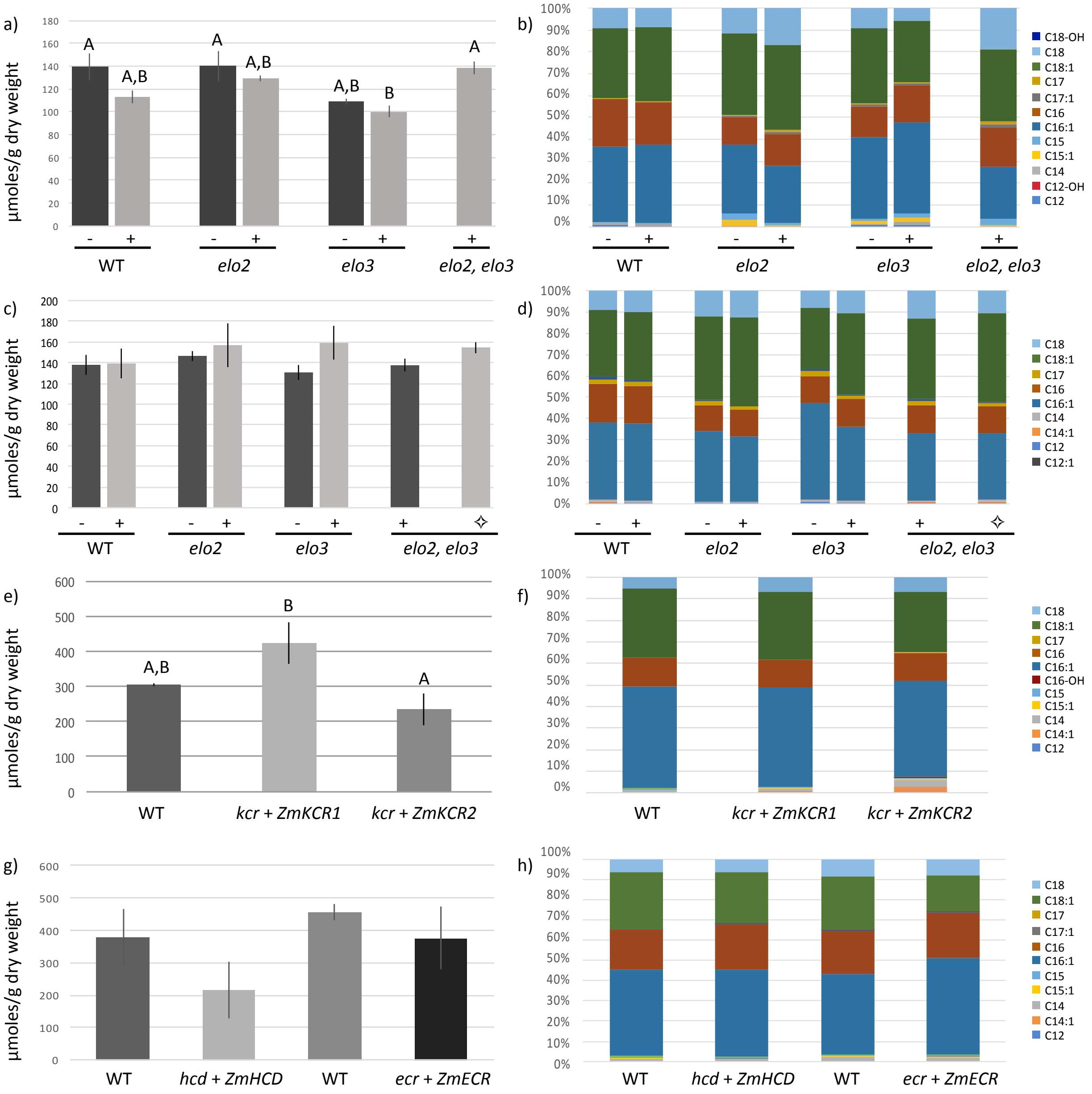

S1 Fig. Total FAS generated fatty acids and product pools. Quantitative totals of FAS products and molar percentage of total FAS product pools for **a, b)** *ZmKCS4* (n=6), **c, d)** *ZmELO1* (n=6) where (◇) indicates the presence of the maintenance plasmid (P_{ELO3}-*ELO3*), **e, f)** *ZmKCR1* and *ZmKCR2* (n=3), and **g, h)** *ZmHCD* (n=8 for control, n=10 for complementing strain) and *ZmECR* (n=8 for control, n=11 for complementing strain). Differing letters indicate statistically significantly different yields based on Tukey HSD (p value<0.05). All strains were analyzed using GC-MS except *ZmELO1,* which was analyzed by GC-FID. Yeast strain is indicated under graphs, were (-/+) indicated the absence and presence of the maize gene respectively for **a-d**.
