# Supplementary material for: A single-cell platform for reconstituting and characterizing fatty acid elongase component enzymes
Source: PLoS One. 2019 Mar 11;14(3):e0213620. doi: 10.1371/journal.pone.0213620 (PMC6411113; doi:10.1371/journal.pone.0213620)
Supplement: S2 Fig — a, c, e, g) WT with empty vector (pYX043, n = 3) and WT with ZmKCR1 (n = 4). a) Quantitative totals of FAS products; b) Molar percentage of total FAS product pools; c) Quantitative totals of VLCFAs; and d) Molar percentages of totally VLCFAs product pools. b, d, f, h) WT with empty vector (pYES2) and WT with ZmKCR2 (n = 5). b) Quantitative totals of FAS products; d) Molar percentage of total FAS product pools; f) Quantitative totals of VLCFAs; and h) Molar percentages of total VLCFA product pools. (PDF) [file pone.0213620.s002.pdf]

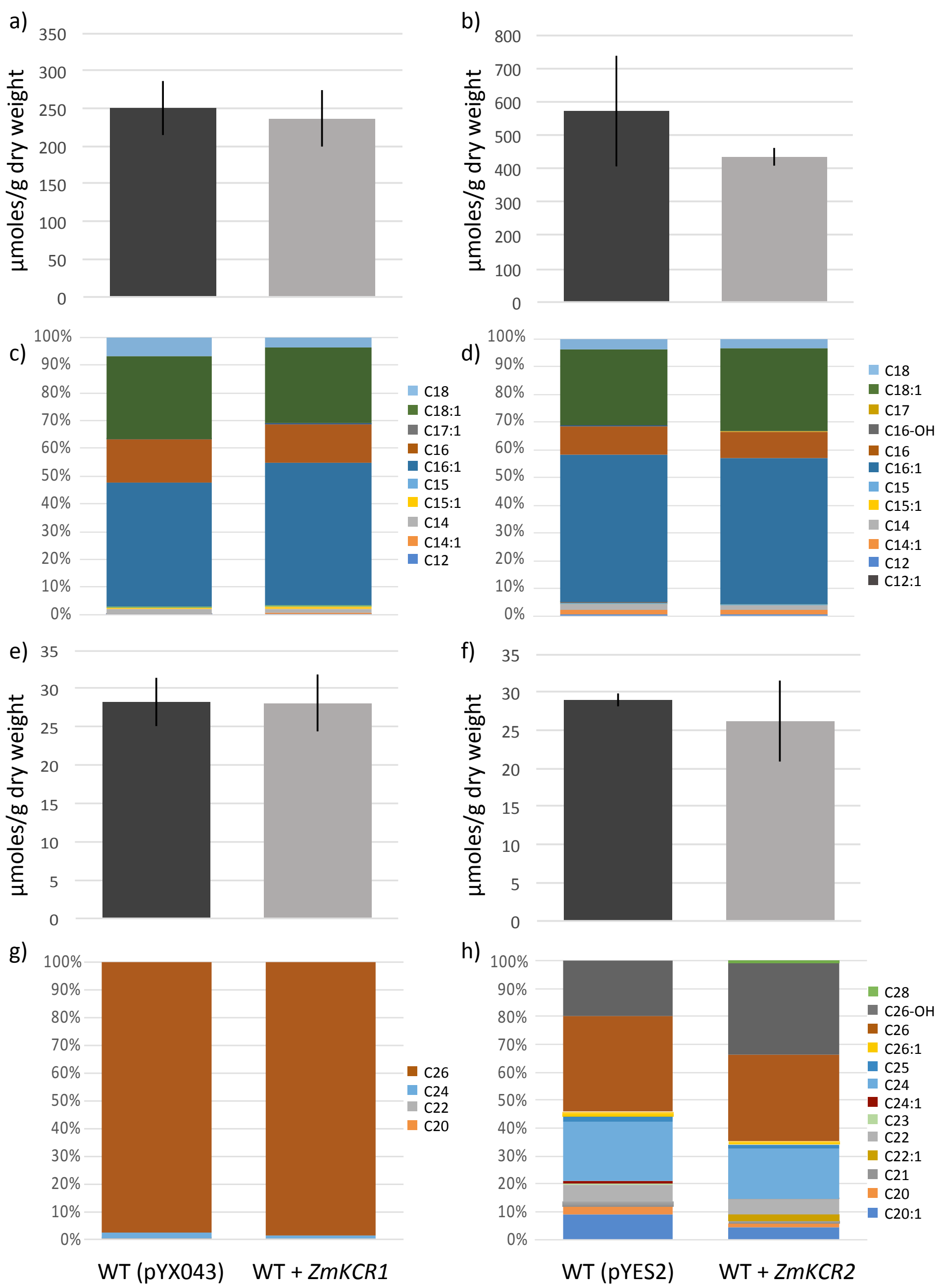

S2 Fig. Total FAS and FAE generated fatty acids and product pools for WT and WT with *ZmKCR1* or *ZmKCR2*. a, c, e, g) WT with empty vector (pYX043, n=3) and WT with *ZmKCR1* (n=4). **a)** Quantitative totals of FAS products; **b)** Molar percentage of total FAS product pools; **c)** Quantitative totals of VLCFAs; and **d)** Molar percentages of totally VLCFAs product pools. **b, d, f, h)** WT with empty vector (pYES2) and WT with *ZmKCR2* (n=5). **b)** Quantitative totals of FAS products; **d)** Molar percentage of total FAS product pools; **f)** Quantitative totals of VLCFAs; and **h)** Molar percentages of total VLCFA product pools.
