# Supplementary material for: A single-cell platform for reconstituting and characterizing fatty acid elongase component enzymes
Source: PLoS One. 2019 Mar 11;14(3):e0213620. doi: 10.1371/journal.pone.0213620 (PMC6411113; doi:10.1371/journal.pone.0213620)
Supplement: S3 Fig — a) Quantitative totals of FAS products; b) Molar percentage of total FAS product pools; c) Quantitative totals of VLCFAs; and d) Molar percentages of totally VLCFAs product pools. Strains were analyzed with replicates of n = 4 for WT with the empty vector (pYX043) and n = 5 for WT with ZmECR. (PDF) [file pone.0213620.s003.pdf]

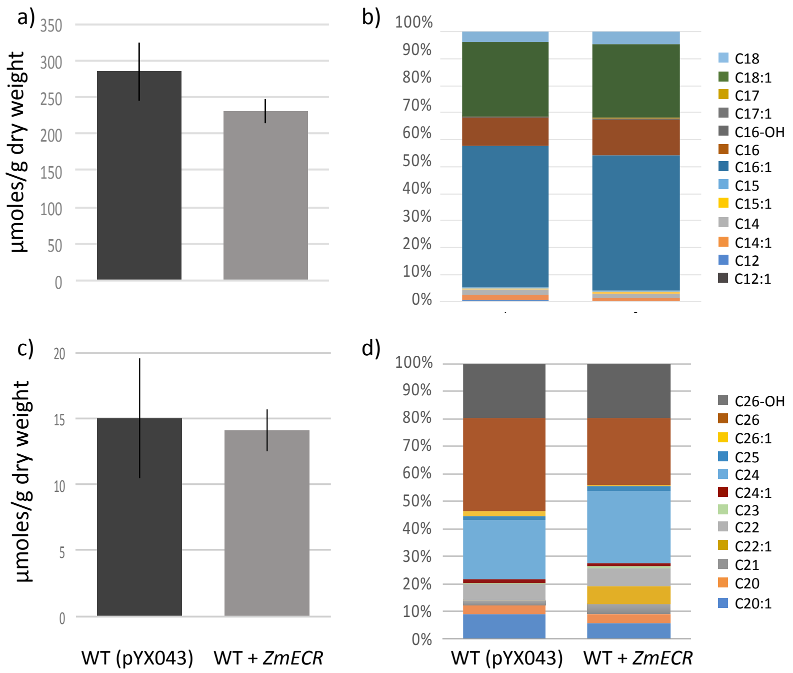

S3 Fig. Total FAS and FAE generated fatty acids and product pools for WT with the empty vector (pYX043) and WT with *ZmECR*. **a)** Quantitative totals of FAS products; **b)** Molar percentage of total FAS product pools; **c)** Quantitative totals of VLCFAs; and **d)** Molar percentages of totally VLCFAs product pools. Strains were analyzed with replicates of n=4 for WT with the empty vector (pYX043) and n=5 for WT with *ZmECR*.
