# Supplementary material for: A single-cell platform for reconstituting and characterizing fatty acid elongase component enzymes
Source: PLoS One. 2019 Mar 11;14(3):e0213620. doi: 10.1371/journal.pone.0213620 (PMC6411113; doi:10.1371/journal.pone.0213620)
Supplement: S1 Table — (PDF) [file pone.0213620.s004.pdf]

**S1 Table. List of plasmids used within this study along with relevant characteristics.**

| **Plasmids used within this work** | | |
|---|---|---|
| **Plasmid** | **Relevent Characteristic(s)** | **Source or Reference** |
| pENTR™ | $Km^r$ | Invitrogen |
| pAG423 | *HIS3*, 2μ, *GAL1* | Alberti, Gitler, and Lindquist, 2007. |
| pAG424 | *TRP1*, 2μ, *GAL1* | Alberti, Gitler, and Lindquist, 2007. |
| pAG426 | *URA3*, 2μ, *GAL1* | Alberti, Gitler, and Lindquist, 2007. |
| pAG416 | *URA3*, CEN, GPD | Alberti, Gitler, and Lindquist, 2007. |
| Modified pAG416 $P_{ELO3}$ | *URA*3, CEN, $P_{ELO3}$ | This work |
| pYES2 | *URA3*, 2μ, *GAL1* | Invitrogen |
| pYX043 | *LEU2*, INT, *GAL1* | Gifted by Alan Meyers Lab |
