# Supplementary material for: A single-cell platform for reconstituting and characterizing fatty acid elongase component enzymes
Source: PLoS One. 2019 Mar 11;14(3):e0213620. doi: 10.1371/journal.pone.0213620 (PMC6411113; doi:10.1371/journal.pone.0213620)
Supplement: S2 Table — (PDF) [file pone.0213620.s005.pdf]

S2 Table. **Primers used to construct FAE reconstitution plasmids and yeast strains.**

Primers Sequences

Primer Name	Primer Sequence (5' to 3')
ZmKCS4-GatF	CACCATGGACGGAGTCTCCGCC
ZmKCS4-GatR	CTATTGCTGCGTGGGGAAG
ZmHCD-GatF	CACCATGGCGGGCGTCGGCT
ZmHCD-GatR	AGCGGCATGATGATGCCAAGCAAG
ZmECR-GatF	CACCATGAAGGTCACGGTCG
ZmECR-GatR	CTTCGCCTCTTCACAGGAACG
ECRcorrA2T	CTTCTTCAGACGGCTGTGCTTGCCGAGCG
ZmELO1-GatF	CACCATGGCGGCCGCGTC
ZmELO1-GatR	TCATTCCGCTTTACCCTTGTTGGCCT
