# Supplementary material for: A single-cell platform for reconstituting and characterizing fatty acid elongase component enzymes
Source: PLoS One. 2019 Mar 11;14(3):e0213620. doi: 10.1371/journal.pone.0213620 (PMC6411113; doi:10.1371/journal.pone.0213620)
Supplement: S3 Table — (PDF) [file pone.0213620.s006.pdf]

S3 Table. Yeast genotype and strain information for this study.

**Yeast Strains**				
**Strain**	**Accession**	**Description**	**(Relevent) Genotype**	**Source**
BY4741 (WT)		BY4741; Mat a Wild Type	BY4741: Mat a; his3D1; leu2D0; met15D0; ura3D0	Open Biosystems
BY4742 (WT)		BY4742; Mat α Wild Type	BY4742: Mat α; his3D1; leu2D0; lys2D0; ura3D0	Open Biosystems
D273		D273; Mat α Wild Type	D273: Mat α; his3D1; leu2D0; ura3D0	Gifted by Alan Meyers Lab
BY4743 (WT)		BY4743; Mat a/α Wild Type	BY4743: Mat a/α; his3D1/his3D1; leu2D0/leu2D0; LYS2/lys2D0; met15D0/MET15; ura3D0/ura3D0	Open Biosystems
scelo2	ELO2, FEN1, YCR034w	Mat a haploid knockout strain	BY4741: Mat a; his3D1; leu2D0; met15D0; ura3D0; YCR034w::kanMX4	Open Biosystems
scelo3	ELO3, SUR4, YLR372w	Mat a haploid knockout strain	BY4741: Mat a; his3D1; leu2D0; met15D0; ura3D0; YLR372w::kanMX4	Open Biosystems
scelo2, scelo3	YCR034w, YLR372w	Heterozygous diploid knockout strain	BY4743: Mat a/α; his3D1/ his3D1; leu2D0/ leu2D0; lys2D0/LYS2; MET15/met15D0; ura3D0/ ura3D0; YCR034w::kanMX4; YLR372w::kanMX4	This Work
sckcr	IFA38, YBR159w	Heterozygous diploid knockout strain	CEN.RO16: CEN.PK; Mat a/alpha: his3D1/ his3D1; leu2D0/ leu2D0; lys2D0/LYS2; MET15/met15D0: ura3D0/ ura3D0; YBR159w::kanMX4/YBR159w	Open Biosystems
schcd	PHS1, YJL097w	Heterozygous diploid knockout strain	BY4743: Mat a/α; his3D1/ his3D1; leu2D0/ leu2D0; lys2D0/LYS2; MET15/met15D0; ura3D0/ ura3D0; YJL097w::kanMX4/YJL097w	Open Biosystems
scecr	TSC13, YDL015c	Heterozygous diploid knockout strain	WDAM006(HE): W303; Mat a/α; ura3-1/ ura3-1; his3-11/ his3-11; leu2-3_112/ leu2-3_112; trp1D2/ trp1D2; ade2-1/ade2-1; can1-100/can1-100; YDL015c(4,702)::kanMX4/YDL015c	Open Biosystems
ZmKCS4/WT	AFW81175.1; GRMZM2G393897	Overexpression of *ZmKCS4* in the WT background	BY4741: Mat a; his3D1; leu2D0; met15D0; ura3D0::URA3/ZmKCS4	This Work
ZmKCS4/*scelo2*		Single component insertion of *ZmKCS4* in the *scelo2* background	BY4741: Mat a; his3D1; leu2D0; met15D0; ura3D0::URA3/ZmKCS4; YCR034w::kanMX4	This Work
ZmKCS4/*scelo3*		Single component insertionof *ZmKCS4* in the *scelo3* background	BY4741: Mat a; his3D1; leu2D0; met15D0; ura3D0::URA3/ZmKCS; YLR372w::kanMX4	This Work
ZmKCS4/*scelo2,* s*celo3*		*ZmKCS4* complementing the *elo2, elo3* double knockout	Sporulated BY4743: his3D1; leu2D0; met15D0; ura3D0::URA3/ZmKCS4; YCR034w::kanMX4; YLR372w::kanMX4	This Work
ZmELO1/WT	CM007647.1; GRMZM2G037152	Overexpression of *ZmELO1* in the WT background	BY4741: Mat a; his3D1::HIS3/ZmELO1; leu2D0; met15D0; ura3D0	This Work
ZmELO1/*scelo2*		Single component insertion of *ZmELO1* in the *scelo2* background	BY4741: Mat a; his3D1::HIS3/ZmELO1; leu2D0; met15D0; ura3D0; YCR034w::kanMX4	This Work
ZmELO1/*scelo3*		Single component insertion of *ZmELO1* in the s*celo3* background	BY4741: Mat a; his3D1::HIS3/ZmELO1; leu2D0; met15D0; ura3D0; YLR372w::kanMX4	This Work
ZmELO1/*scelo2, scelo3*		*ZmELO1* complementing the *scelo2,* s*celo3* double knockout	Sporulated BY4743: his3D1::HIS3/ZmELO1; leu2D0; met15D0; ura3D0; YCR034w::kanMX4; YLR372w::kanMX4	This Work
ScELO3/scelo2, scelo3		*ScELO3* (pAG413, low-copy, *URA3*, P_{ELO3}-*ELO3)*	Sporulated BY4743: his3D1; leu2D0; met15D0; ura3D0::URA3/P_{ELO3}-ELO3; YCR034w::kanMX4; YLR372w::kanMX4	This Work
ZmKCR1/*sckcr*		Overexpression of *ZmKCR1* in the s*ckcr* background	Sporulated CEN.RO16: CEN.PK; his3D1; leu2D0::LEU2/ZmKCR1; ura3D0; kanMX4/YBR159w	This Work
ZmKCR2/*sckcr*		Overexpression of *ZmKCR2* in the *sckcr* background	Sporulated CEN.RO16: CEN.PK; his3D1; leu2D0::LEU2/ZmKCR2; ura3D0; kanMX4/YBR159w	This Work
ZmKCR1/WT		Overexpression of *ZmKCR1* in the WT background	D273: Mat α; his3D1; leu2D0::LEU2/ZmKCR1; ura3D0	This Work
ZmKCR2/WT		Overexpression of *ZmKCR2* in the WT background	D273: Mat α; his3D1; leu2D0::LEU2/ZmKCR2; ura3D0	This Work
ZmHCD/WT		Overexpression of *ZmHCD* in the WT background	Sporulated BY4743: his3D1::HIS3/ZmHCD: leu2D0; ura3D0	This Work
ZmHCD/*schcd*		Overexpression of *ZmHCD* in the s*chcd* background	Sporulated BY4743: his3D1::HIS3/ZmHCD; leu2D0; ura3D0; kanMX4/YJL097w	This Work
ZmECR/WT		Overexpression of *ZmECR* in the WT background	WDAM006(HE): W303; ura3-1; his3-11; leu2-3_112; trp1D2::TRP1/ZmECR; ade2-1; can1-100	This Work
ZmECR/*scecr*		Overexpression of *ZmECR* in the *scecr* background	WDAM006(HE): W303; ura3-1; his3-11; leu2-3_112; trp1D2::TRP1/ZmECR; ade2-1; can1-100; kanMX4/YDL015c	This Work
